# Evidence that endogenous formaldehyde produces immunogenic and atherogenic adduct epitopes

**DOI:** 10.1038/s41598-017-11289-8

**Published:** 2017-09-07

**Authors:** Jun Nakamura, Takasumi Shimomoto, Leonard B. Collins, Darcy W. Holley, Zhenfa Zhang, Jenna M. Barbee, Vyom Sharma, Xu Tian, Tomohiro Kondo, Koji Uchida, Xianwen Yi, Diana O. Perkins, Monte S. Willis, Avram Gold, Scott J. Bultman

**Affiliations:** 1 Department of Environmental Sciences and Engineering, University of North Carolina at Chapel Hill, Chapel Hill, NC USA; 2 Department of Genetics, University of North Carolina at Chapel Hill, Chapel Hill, NC USA; 3 Department of Psychiatry, University of North Carolina at Chapel Hill, Chapel Hill, NC USA; 4 Lineberger Comprehensive Cancer Center, University of North Carolina at Chapel Hill, Chapel Hill, NC USA; 5 Department of Pathology & Laboratory Medicine, University of North Carolina at Chapel Hill, Chapel Hill, NC USA; 6 McAllister Heart Institute, University of North Carolina at Chapel Hill, Chapel Hill, NC USA; 7 Laboratory of Laboratory Animal Science, Graduate School of Life and Environmental Biosciences, Osaka Prefecture University, Izumisano, Osaka Japan; 8 School of Bioagricultural Sciences, Nagoya University, Nagoya, Japan

## Abstract

Endogenous formaldehyde is abundantly present in our bodies, at around 100 µM under normal conditions. While such high steady state levels of formaldehyde may be derived by enzymatic reactions including oxidative demethylation/deamination and myeloperoxidation, it is unclear whether endogenous formaldehyde can initiate and/or promote diseases in humans. Here, we show that fluorescent malondialdehyde-formaldehyde (M2FA)-lysine adducts are immunogenic without adjuvants in mice. Natural antibody titers against M2FA are elevated in atherosclerosis-prone mice. Staining with an antibody against M2FA demonstrated that M2FA is present in plaque found on the aortic valve of *ApoE* ^−/−^ mice. To mimic inflammation during atherogenesis, human myeloperoxidase was incubated with glycine, H_2_O_2_, malondialdehyde, and a lysine analog in PBS at a physiological temperature, which resulted in M2FA generation. These results strongly suggest that the 1,4-dihydropyridine-type of lysine adducts observed in atherosclerosis lesions are likely produced by endogenous formaldehyde and malondialdehyde with lysine. These highly fluorescent M2FA adducts may play important roles in human inflammatory and degenerative diseases.

## Introduction

**Figure 1:**
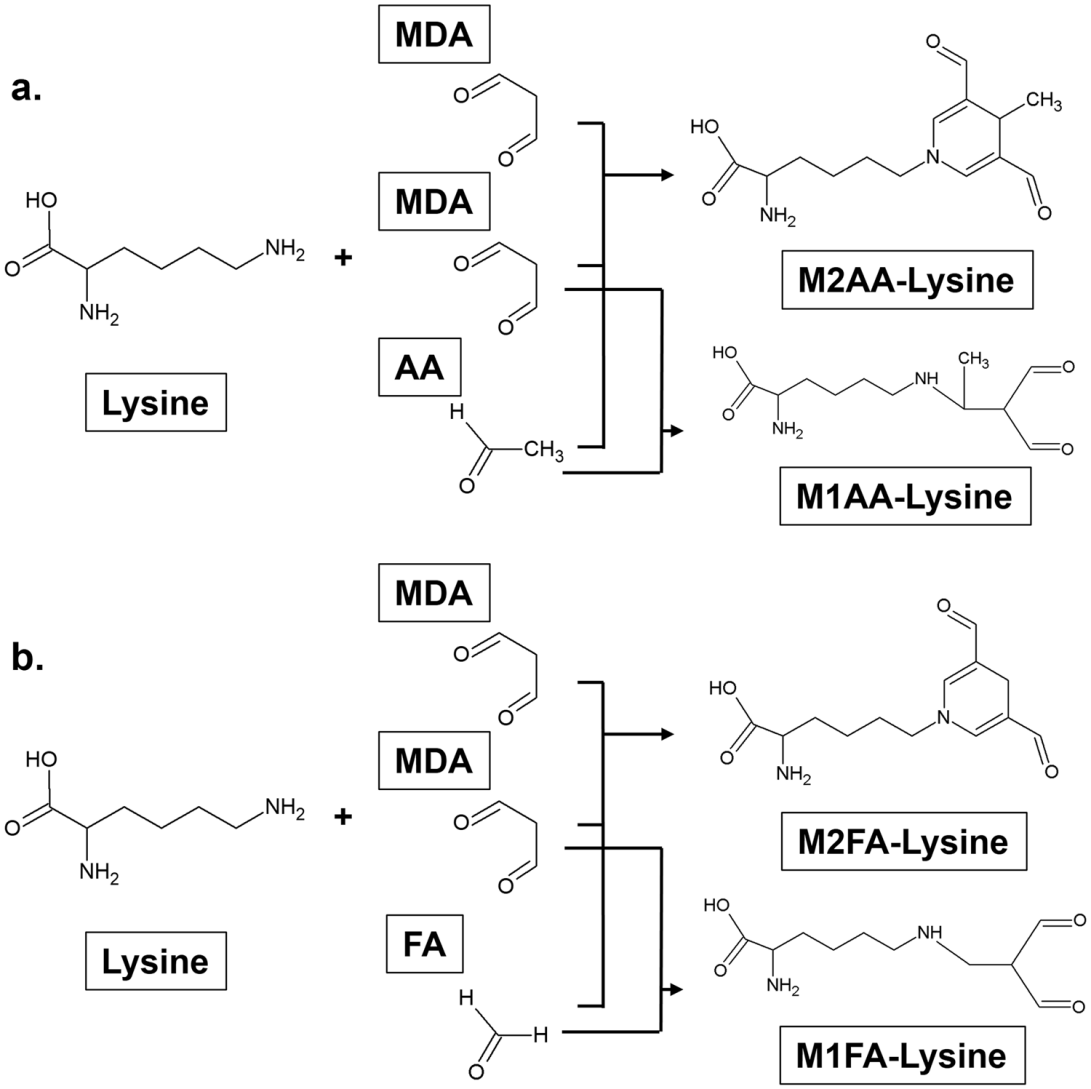
Structure of the M2AA-lysine, M1AA-lysine, M2FA-lysine, and M1FA-lysine adducts. (**a**) 1,4-dihydropyridine-type M2AA-Lys adduct is formed by a reaction between AA and two equivalents of MDA with a primary amine, usually at the ε-position amino moiety of a Lys residue on the target protein. M1AA-lysine adduct is produced by a reaction between AA and MDA in the presence of Lys (**b**) M2FA-Lys adduct is formed by a reaction between FA and two equivalents of MDA with Lys. M1FA-lysine adduct may be produced by a reaction between MA and MDA in the presence of Lys.

Oxidative stress plays an important role in the initiation and progression of various human diseases. Oxygen free radicals attack DNA, protein, and lipids, leading to different types of post-translational modifications of cellular macromolecules. For example, unsaturated lipid cell membranes are readily oxidized, resulting in lipid peroxidation and the formation of alkanes, alcohols, hydroperoxides, and highly reactive aldehydes. The reactive aldehydes can further modify cellular macromolecules to form specific adducts, modifying lysine (Lys) groups which are frequent targets of post-translational modifications. One of the most abundant endogenous aldehydes produced by lipid peroxidation is malondialdehyde (MDA)^1^, which is commonly increased in human disease, including neurodegenerative and cardiovascular diseases, diabetes, and age-related macular degeneration^2, 3^. Interestingly, many of these diseases are associated with the immune response. Although MDA is less reactive compared to α, β-unsaturated aldehydes produced by lipid peroxidation, MDA has the unique property of modifying macromolecules through (1) simple mono-adduction^4^, (2) crosslinkage^5^, and (3) hybrid adduct formation in the presence of other aldehydes^4^. One example of these hybrid adducts is the 1,4-dihydropyridine-type MDA-acetaldehyde (AA)-Lys adduct (hereafter referred to as M2AA) (Fig. 1a), which consists of two molecules of MDA, its degradation product AA, and Lys^6^. M2AA has also been referred to as MAA in many previously published articles. M2AA-modified low-density lipoprotein (LDL), which is a form of oxidized LDL (oxLDL), has been implicated in atherogenesis^6–8^. M2AA-Lys adducts have been reported to be highly stable^9^, toxic^10^, pro-inflammatory^11^ and profibrogenic^12, 13^. Repeated immunization with M2AA-modified protein induces robust antibody production even in the absence of adjuvant^14^. Thus, M2AA-Lys adducts have been proposed to be an immunodominant epitope and one of the most potent atherogenic protein adducts caused by lipid peroxidation^6, 7^. As such, M2AA adducts appear to play critical roles in atherogenesis^6^.

In peripheral blood, however, MDA exists at low concentrations^15, 16^; accordingly, the levels of AA derived from MDA may be even lower. Thus, likelihood of the generation of the high levels of M2AA in human tissues in oxidative stress conditions has been under debate^17^. In contrast to AA, significant amounts of formaldehyde (FA) exist in the blood and tissues under physiological conditions^18, 19^. Although the major source of endogenous FA formation is unknown, it has been seen that FA is produced in the human body via enzymatic oxidative demethylation of DNA, RNA, and protein^20^, oxidative deamination of methylamine, as well as in the myeloperoxidase (MPO) reaction of activated neutrophils and monocytes^21^. FA is known to be two orders of magnitude more reactive than AA towards protein and DNA^22^. These findings point to the possibility that MDA produced from lipid peroxidation may react with endogenous FA, leading to MDA-FA (M2FA) protein adducts (Fig. 1b). Furthermore, most previously published reports on the subject of M2AA epitope detection have utilized antibody-based assays; therefore, there is concern that these antibodies could recognize epitopes that are structurally similar to M2AA. Due to the chemical and structural similarities between FA and AA, some of the epitopes previously detected by M2AA antibodies may be M2FA protein adducts. In this study, we successfully synthesized M2FA *via* a reaction between Lys, MDA, and FA. IgG and IgM in blood collected from human and mice recognized the purified M2FA epitope, and titers of these antibodies appear to increase under metabolic disease.

## Results

### Synthesis of N^ε^-M2FA-Lys and N^ε^-M2FA-aminocaproic acid (N^ε^-M2FA-6ACA)

**Figure 2:**
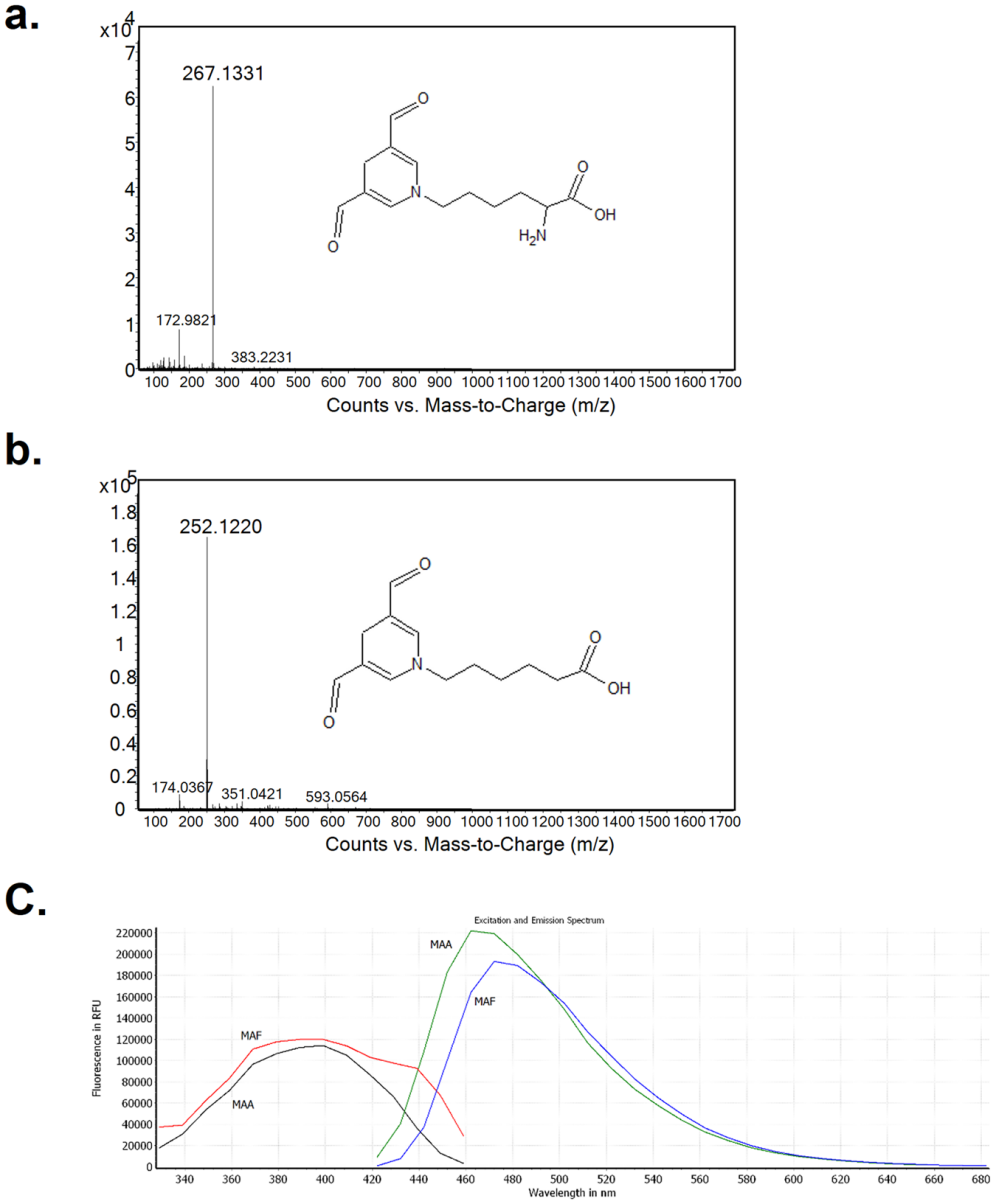
The full scan mass spectra of M2FA-lysine and M2FA-6ACA and fluorescence spectrum of M2FA- and M2AA-6ACA. (**a**) The background-subtracted full scan mass spectrum shows the protonated molecular ion for M2AA-lysine at *m/z* 267.1331. (**b**) The background-subtracted full scan mass spectrum shows the protonated molecular ion of M2AA-6ACA at *m/z* 252.1220. (**c**) Fluorescence spectrum of M2FA- and M2AA-6ACA.

We first incubated FA and MDA with *N* ^α^-(*t*-Butoxycarbonyl)-L-Lys (*N* ^α^-Boc-L-Lys) in PBS at 37 °C. A gradual change in the color of the reactant was observed with increasing incubation time. The reaction mixture was purified via HPLC monitored by UV at 260 nm, and fractions with strong fluorescence (400 nm excitation/460 nm emission) were collected (identical conditions used for detection of M2AA-6ACA^23^). Following deprotection of Boc and HPLC purification, the M2FA-Lys adduct was characterized by LC-MS and NMR (Figs 2a and S1b). As an alternative to the two-step HPLC purification method for M2FA-Lys synthesis, the analog *N* ^ε^-M2FA-aminocaproic acid (M2FA-6ACA) was synthesized by the reaction of 6-ACA with MDA and FA (Fig. S2a). 6-aminocaproic acid (6-ACA) is an analog of Lys that lacks the α-amino group. M2FA-6ACA was purified by HPLC, and its identity and purity were confirmed by LC-MS and ^1^H NMR (Figs 2b and S2b).

### Fluorescence properties of pure M2FA and M2AA adducts

M2FA-Lys and M2FA-6ACA adducts exhibit fluorescence similar to the 1,4-dihydropyridine fluorophore of the M2AA-Lys analog^24–27^. In this study, to characterize the similarity between M2FA-6ACA and M2AA-6ACA, we compared the extent of UV absorbance and fluorescence properties between the two adducts. M2FA-6ACA and M2AA-6ACA have excitation/emission maxima at 389/472 nm and 399/462 nm, respectively (Fig. 2c). These values are in line with previous results reported for M2AA-Lys analogs^24^. Although UV absorbances of the two molecules were similar, the M2FA was a weaker fluorophore than M2AA (Fig. S4).

### Anti-M2FA antibody reactivity toward purified M2FA-6ACA and M2AA-6ACA

**Figure 3:**
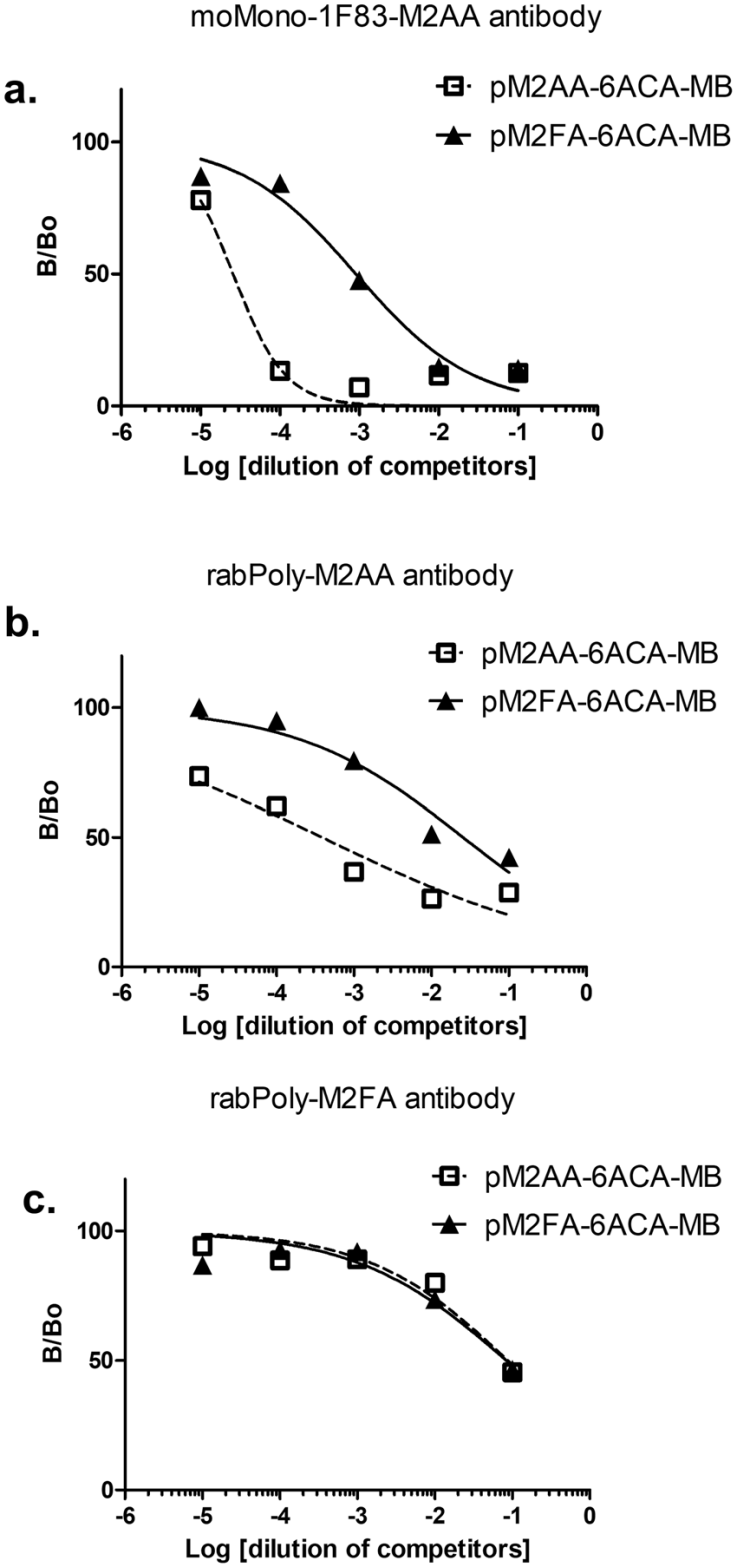
Competitive inhibition of M2FA- and M2AA-MB in competitive ELISA. The relative ability of M2FA- and M2AA MB to inhibit antibody. Purified anti-M2AA and M2FA antibodies (**a**) moMono-1F83-M2AA Ab; (**b**) rabPoly-M2AA Ab; (**c**) rabPoly-M2FA Ab) were preincubated with the indicated competitors and controls prior to detection of IgG binding to plated M2FA-6ACA-BSA. Data are shown as B/B_0_ of triplicates for a representative experiment.

Using M2FA-6ACA-bovine serum albumin (BSA), we raised a rabbit polyclonal antibody against M2FA. The serum obtained from rabbits immunized with M2FA-6ACA-BSA in the presence of adjuvant showed high anti-M2FA IgG titer (Fig. S5). Interestingly, serum from pre-immunized rabbits demonstrated the presence of antibodies against M2FA. After affinity purification of the IgG antibody, the anti-M2FA polyclonal antibody was compared to anti-M2AA antibodies with regards to specificity by competitive ELISA assay using M2FA-6ACA- and M2AA-6ACA-magnetic beads (MB). Both the anti-M2AA monoclonal (1F83) and anti-M2AA polyclonal antibodies showed 2 to 3 orders of magnitude higher response to M2AA-6ACA than M2FA-6ACA (Fig. 3a,b). In contrast, the anti-M2FA polyclonal antibody recognized both epitopes at similar levels (Fig. 3c).

### Immunogenicity of M2FA-Lys-BSA

**Figure 4:**
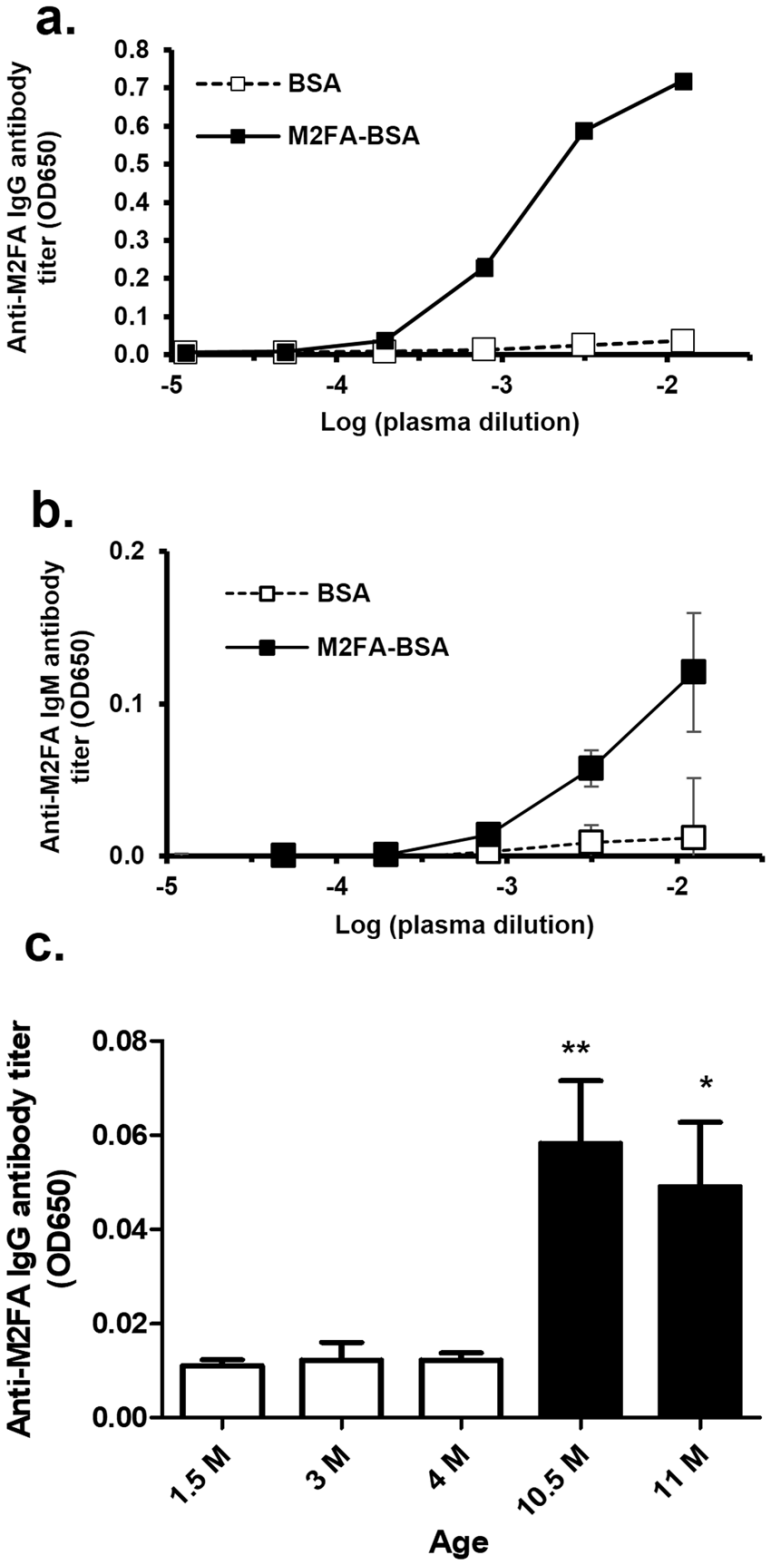
The immunogenicity of M2FA-lysine-BSA in the absence of adjuvant and anti-M2FA antibody titers in intact mice. C57BL/6 mice were injected i.p. with M2FA-lysine-BSA or BSA in the absence of adjuvant. The antibody titers of IgG (**a**) and IgM (**b**) against M2FA-lysine were detected using MFA-6ACA-KLH-coated plates. The anti-M2AA antibody titers were clearly increased in M2FA-lysine-BSA-immunized mice compared to the controls (BSA-treated mice). Values are mean and SD. (**c**) Intact female C57BL/6 mice (n = 4 or 5 per group) with different ages showed significantly different anti-M2FA IgG titers. Values are mean and SD. (*p < 0.05; between 1.5 M/3 M vs 11 M; **p < 0.01: 1.5 M/3 M/4 M vs 10.5 M).

To evaluate whether M2FA-Lys adducts are immunogenic in the absence of adjuvant, C57BL/6 mice were treated intraperitoneally with either M2FA-Lys-BSA or BSA. The titers of IgG and IgM antibodies directed against M2FA-Lys, which were detected using M2FA-6ACA-keyhole limpet hemocyanin (KLH)-coated plates, showed a clear increase in M2FA-BSA-immunized mice as compared to their controls (BSA-treated mice) (Fig. 4a and b). Comparative results have been reported in mice treated with crude M2AA-BSA without adjuvant^14^.

### Antibodies against M2FA are present in intact mice at different ages

IgM antibodies against lipid peroxidation-derived modifications exist in mice and humans under normal conditions^28^. Interestingly, ~30% of all natural antibodies bind to MDA as well as M2AA-modified protein. The natural antibodies might be predicted to increase in aged mice even under healthy conditions due to long-term physiological levels of oxidative stress. However, as mentioned above, previous studies measuring M2AA antibody titers were based on ELISAs using crude epitopes prepared by simple incubation of protein and MDA and/or AA. To identify if specific M2FA antibodies were induced in intact mice, we assayed serum with either pM2FA-specific ELISAs to determine the amount of antigen specific-IgG levels in C57BL/6 mice. Both IgG and IgM titers were detected in specific pathogen free, female C57BL6 mice at 1.5- to 4-month old (Fig. 4c). When comparing the antibody titers to those in older mice, ~4 to 5 times higher titers were observed in female 11-month-old mice. To our best knowledge, this is the first evidence of the presence of much higher titers of antibody against 1,4-dihydropyridine-type adducts in aged mice, suggesting either an accumulation of epitopes in tissues or an elevation of the immune response to the target epitopes in aged mice.

### Anti-M2FA antibody titer in the serum of atherosclerosis-prone *ApoE*^−/−^ mice

**Figure 5:**
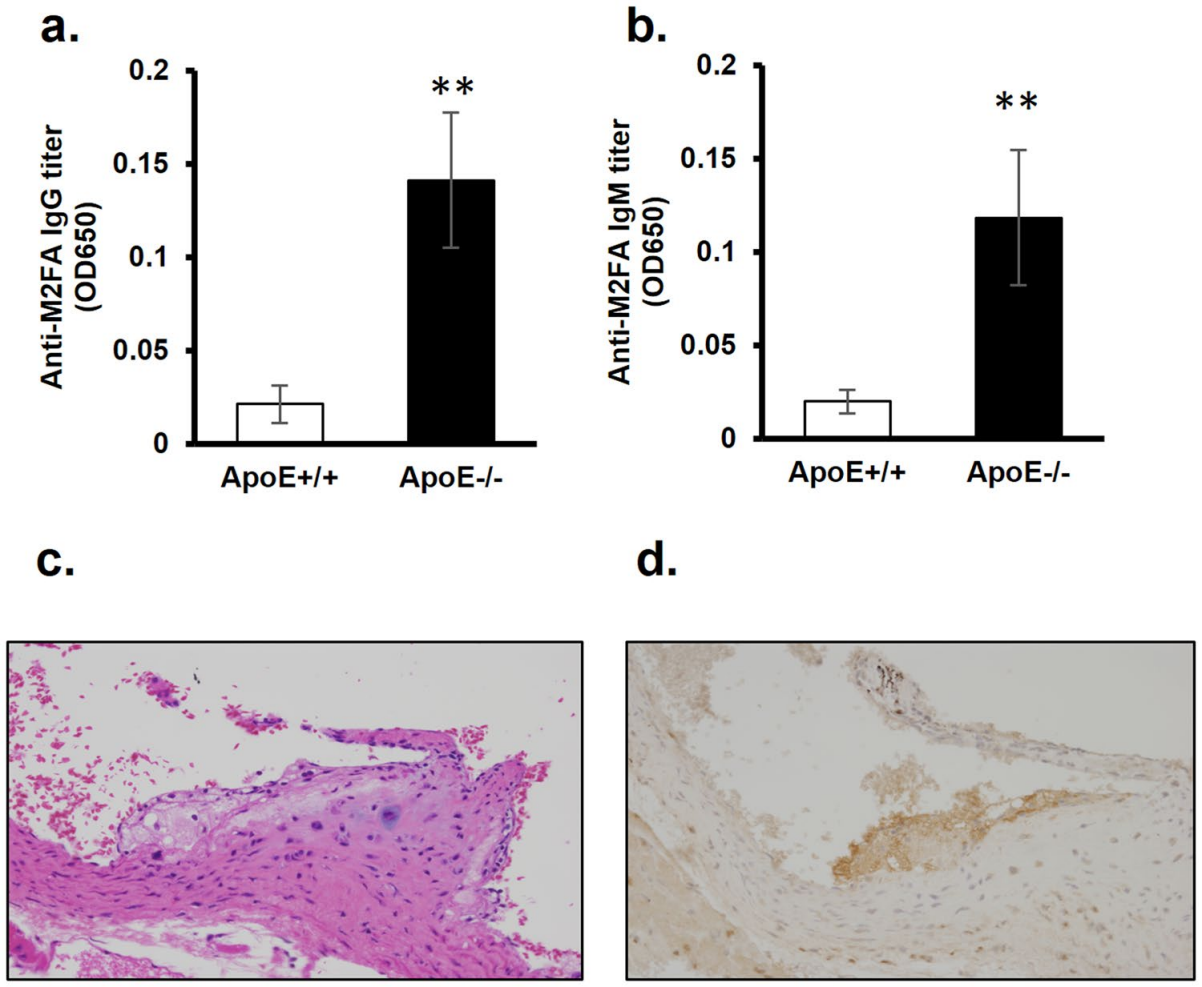
Serum anti-M2FA IgG and IgM antibody levels in *wild-type* and *ApoE* ^−/−^ mice and immunohistochemical detection of M2FA-epitopes in heart valve of *ApoE* ^−/−^ mice. The anti-M2FA IgG (**a**) and IgM (**b**) antibody levels showed significant differences between *wild-type* and *ApoE* ^−/−^ mice with the M2FA-6ACA-BSA ELISAs (**p < 0.01). Representative H&E (**c**) and M2FA immunohistochemistry (**d**) sections of plaques in heart valve of *ApoE* ^−/−^ mice at 5 month old fed with normal diet. M2FA-lysine in the plaque was stained with rabPoly-M2FA Ab.

Increased levels of IgG and/or IgM antibodies against MDA-modified LDL have been reported in serum of *ApoE* ^−/−^ mice fed with both a high fat diet^29^ and normal chow^30^ compared to wild-type mice. Due to strong immunogenicity of M2AA adducts, the increased antibody titers against MDA-modified LDL may be largely attributable to anti-M2AA antibodies in the *ApoE* ^−/−^ mice. Indeed, our independent study demonstrated a significant increase in serum IgG and IgM titers against the pure M2AA epitope^23^. Since we found that M2FA is highly immunogenic in C57BL/6 mice without adjuvant, antibodies to M2FA could be expected to increase as endogenous M2FA epitopes accumulate in the atherosclerosis-prone mice. Thus, we investigated whether serum levels of IgG and IgM antibodies against M2FA are increased in atherosclerosis-prone *ApoE* ^−/−^ mice fed with a normal diet. In blood serum collected from 13 week *ApoE* ^−/−^ mice, IgG and IgM antibody titers were significantly higher in *ApoE* ^−/−^ mice than those in wild type mice (Fig. 5a,b).

### Atherosclerosis plaque detection using anti-M2FA antibody

We next addressed whether the polyclonal antibody against M2FA recognizes atherosclerosis plaque in *ApoE* ^−/−^ mice. Since the widely-used fixative, paraformaldehyde, contains a very high concentration of FA, we instead utilized zinc-based fixative to avoid artifactual formation of M2FA during tissue sample preparation. In addition, we used a zinc-specific dehydration/paraffin embedding system for eliminating FA contamination. Figure 5c clearly shows the presence of M2FA -positive plaque on the aortic valve in an *ApoE* ^−/−^ mouse using the FA-free fixative method.

### MPO induces M2FA

**Figure 6:**
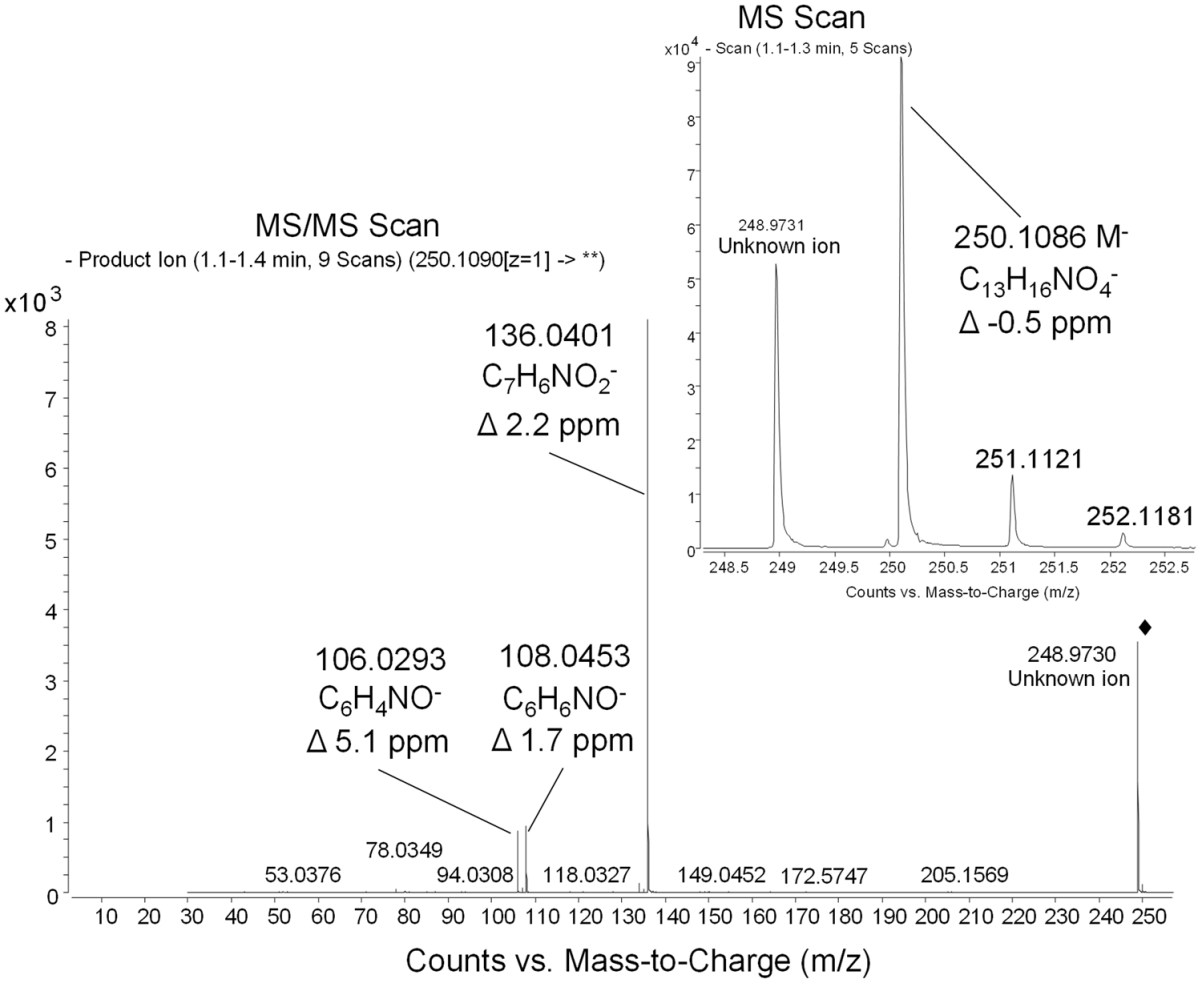
Liquid chromatography−mass spectrometry analyses demonstrating M2FA formation through the MPO-H_2_O_2_-halide system. Glycine, human MPO, H_2_O_2_ were incubated in PBS for 1 hour at 37 °C followed by incubation with MDA and 6-ACA (Lys analog) for 3 days at 37 °C. The reactant was applied to LC-MS analysis. Full scan and ms/ms spectra of M2FA-6ACA were obtained on an Agilent 6520 Accurate Mass Q-TOF in negative mode. Spectra show the intact de-protonated molecular ion at *m/z* 250.1086 (Δ −0.5 ppm) and major fragments at *m/z* 136.0401 (Δ 2.2 ppm), 108.0453 (Δ 1.7 ppm), and 106.0293 (Δ 5.1 ppm). Chemical formulas are proposed for the fragments, and deviations of measured masses to calculated masses for these formulas are all 5 ppm or less. The product ion spectrum was obtained using *m/z* 250.1090 as the precursor and 20 eV for collision energy.

Endogenous FA can be high enough to produce M2FA in the presence of MDA at inflammation sites, such as atherosclerosis lesions. Interestingly, MPO in activated neutrophils, monocytes, and macrophages under inflammation produces an additional burden of reactive aldehydes through the MPO-H_2_O_2_-halide system^31, 32^, which plays a major role in the antimicrobial effects of leukocytes. Previous studies have found that incubation of MPO with H_2_O_2_, chloride ions, and either threonine, serine, and glycine leads to the formation of glycolaldehyde^33^, acrolein^34^, and FA^21^, respectively. Here, we tested whether the MPO-H_2_O_2_-halide system could generate M2FA in the presence of MDA and a Lys analog without supplementing FA to mimic atherosclerosis lesions in the presence of activated monocytes and macrophages. After a one-hour incubation of MPO and H_2_O_2_ in PBS at 37 °C, MDA and 6ACA were further added into the reaction mixture followed by incubation at 37 °C for 3 days. We then collected the M2FA-6ACA peak followed by LC-MS analysis. The fractionated sample contained M2FA-6ACA identified by molecular mass and fragmentation pattern (Fig. 6). The results clearly demonstrate that MPO has the potential to produce M2FA under physiological temperature and pH. The results also strongly suggest that atherosclerosis lesions possess an environment conducive for M2FA production under lipid peroxidation because of likely higher steady-state levels of endogenous FA derived from an MPO-mediated reaction and physiological FA metabolism.

## Discussion

M2AA adducts have been implicated in atherogenesis^6–8^, as well as in various other inflammatory diseases including age-related macular degeneration, rheumatoid arthritis, and diabetes^2, 35, 36^. However, low endogenous levels of AA, which is one of the components necessary to generate M2AA adducts, calls into question its ability to generate high levels of the adducts. Compared to AA, endogenous FA exists at a much higher concentration in the blood, ranging from 27–98 µM under physiological conditions^18, 19^. In the current study, we have shown for the first time that M2FA can be synthesized in a physiological solution containing MDA and FA in a 2:1 molar ratio. ELISAs coated with pure M2FA detected the presence of IgG and IgM antibodies against M2FA in the sera of rabbits and mice. The M2FA ELISA also showed an increase in antibodies against M2FA in aged mice and atherosclerosis-prone mice. The results of this study, in combination with published evidence of the role of endogenous FA in the formation of M2FA (as discussed later), point to the possibility that M2FA could play roles in inflammatory diseases such as atherosclerosis.

The initiation and progression of atherosclerosis involve many different factors including genetics, behavioral factors, environmental chemicals (e.g., PAH, PCBs, and arsenic) and endogenous reactive agents (e.g., acrolein and MDA). It has been shown that MDA and AA react with Lys, resulting in M2AA-Lys adducts, which have been proposed to be an immunodominant epitope and one of the most potent atherogenic protein adducts caused by lipid peroxidation^6, 7^. Due to the very similar structures of M2AA and M2FA (the difference lying in the presence or absence of the methyl moiety the on 1,4-dihydropyridine ring, respectively), we believe that these two analogous Lys adducts share similar biological effects including high stability^9^, toxicity^10^, pro-inflammatory^11^ and profibrogenic^12, 13^ properties, and immunogenicity.

Similar to the strong immunogenicity of M2AA^14^, we found that immunization with pure M2FA-adducted BSA induces robust antibody production even in the absence of adjuvant. In the present study, we also demonstrated a significant increase in IgG and IgM antibodies against M2FA in the atherosclerosis-prone *ApoE* ^−/−^ mice. The atherosclerotic plaques of the *ApoE* ^−/−^ mice positively stained with a polyclonal antibody against M2FA. However, the question as to whether the epitopes in atherosclerosis are M2FA or M2AA adducts still remains. Although the anti-M2AA antibodies tested in this study demonstrated higher immunoreactivity against M2AA compared to M2FA, the anti-M2AA antibodies still exhibited immunoreactivity against M2FA. This introduces the possibility that some of the epitopes identified as M2AA in previous studies may be M2FA adducts. However, the same caveat exists for the current study, wherein the anti-M2FA antibody may be targeting both M2FA and M2AA, due to the cross-reactivity of the anti-M2FA antibody against M2AA. Interestingly, although the mechanism for the generation of high levels of endogenous FA has yet to be identified, one of the mechanisms of generating endogenous FA is an MPO-mediated reaction^21^. It has been shown that the MPO of activated neutrophils and monocytes at sites of inflammation produce toxic FA from glycine^21^. Stimulated, these white blood cells are well known to present at atherosclerosis lesions; therefore, atherosclerosis lesions may exhibit even higher concentrations of endogenous FA than other tissues and plasma. In the present study, we characterized M2FA adduct production by the MPO- H_2_O_2_-halide system in the presence of MDA and a Lys analog in the absence of FA supplementation. Put together, we now propose that M2FA may play important roles in atherogenicity.

Surprisingly, previous studies have reported that ~30% of the natural IgM antibody reacts against both MDA-modified protein and M2AA^28^. A recent paper also demonstrated that relative levels of IgM antibody that can recognize MDA-modified protein is higher in cord blood than that in adults^37^. These IgM antibodies against MDA and M2AA may be autoantibodies against oxidized neo-epitopes in apoptotic cells. Although the mechanism by which embryos generate the natural IgM antibody against oxidized neo-epitopes is not well understood, some of the oxidized neo-epitopes may be derived from apoptotic cells from fetal development or the placenta during the gestational period^37^. IgM antibodies against oxidized neo-epitopes during gestation may increase apoptotic cell clearance during embryogenesis and also inhibit unnecessary inflammation. In the present study, ELISAs using pure M2FA and M2AA epitopes without contamination of MDA-mono-adducts or M1FA adducts (Fig. 1) were able to detect IgM antibodies against these target epitopes in mice, suggesting the existence of a natural antibody against 1,4-dihydropyridine-type Lys adducts, such as M2FA.

In humans and mice, M2FA may be generated as a damage-associated molecular pattern (DAMP), which can potentially cause sterile inflammation. DAMPs are located in damaged tissues (e.g., plaque in atherosclerosis) and are also released from cells undergoing cell death. The increase in IgG antibodies against M2FA in atherosclerosis-prone mice may suggest a consistent production of immunogenic and very stable M2FA adducts as DAMPs in mice and possibly in humans. IgG and IgM antibodies may bind to M2FA-Lys adducts, potentially leading to the protection of sterile inflammation as well as clearance M2FA-modified proteins.

In addition to MPO, semicarbazide-sensitive amine oxidases (SSAOs) deaminate endogenous methylamine, resulting in the formation of FA, H_2_O_2_, and ammonia^38^. In fact, this family of enzymes was independently discovered as a vascular adhesion molecule (VAP-1)^39^. VAP-1/SSAO, which has been found to have both enzymatic and adhesive functions, is present on the surface of vascular endothelium^40^. In the presence of VAP-1/SSAO, leukocytes can adhere to endothelial cells and transmigrate through the endothelium into the tissues^41–44^. This leukocyte trafficking activity requires both the enzymatic and adhesive functions of VAP-1/SSAO^43, 45^. Interestingly, expression of VAP-1 on the endothelium has been found to be increased under inflammation^41–43^, and increased serum VAP-1 released by endothelial cells is positively associated with inflammatory diseases including atherosclerosis^46^. Furthermore, it has been shown that diabetic/atherosclerosis mice treated with a VAP-1/SSAO inhibitor exhibit increases in urinary methylamine and decreases in FA and MDA in the urine and atherosclerotic lesions^47^. One possible mechanism for all of this could be that during leukocyte trafficking, the enzymatic activity of VAP-1/SSAO may result in the generation of FA and H_2_O_2_ by-products, similar to myeloperoxidation. H_2_O_2_ generation, as well as glutathione depletion resulting from increased FA, can lead to oxidative stress and lipid peroxidation, resulting in MDA formation. Based on this reported evidence, in combination with the present study, the combination of VAP-1/SSAO and MPO could synergistically increase production of M2FA at atherosclerosis sites.

While several biologically essential enzymatic reactions are known to produce endogenous FA, leading to surprisingly high steady-state levels of FA in our bodies, the pathophysiological consequence of endogenous FA has not been well-characterized. This study reveals that M2FA can be formed by the reaction between endogenous FA, MDA, and lysine under physiological conditions. These stable M2FA adducts may be an abundant, endogenous 1,4-dihydropyridine-type lysine adduct species. Through these studies, we have shown that M2FA is not only markedly immunogenic but also may be an important mediator of sterile inflammation involved in human diseases (e.g., atherosclerosis, rheumatoid arthritis, and age-related macular degeneration).

## Materials and Methods

### Materials

N^α^-(tert-butoxycarbonyl)-L-Lys (Boc-Lys), ε-aminocaproic acid (6-ACA), AA, FA, BSA, trifluoroacetic acid, 3,3′,5,5′-tetramethylbenzidine (TMB), human MPO, and H_2_O_2_ were purchased from Sigma-Aldrich (St. Louis, MO). Malondialdehyde bis(dimethyl) acetal, Dynabeads M-270 Amine, CarboxyLink Kit, the Imject EDC mcKLH Spin Kit, goat anti-rabbit IgG (H + L) antibody with HRP, goat anti-human IgG/IgM secondary antibody, and goat anti-mouse IgM secondary antibody were obtained from Thermo Scientific (Rockford, IL). Protein A column and autoLDL™ Cholesterol kit were purchased from GE Healthcare Life Sciences (Pittsburgh, PA) and Pointe Scientific (Lincoln Park, MI), respectively. EnVision + Single Reagents anti-mouse/rabbit-HRP were from Dako North America, Inc. (Carpentaria, CA). The anti-M2AA mouse monoclonal antibody (1F83)^24^ and rabbit polyclonal antibody^48^ were provided by Drs. Koji Uchida (Nagoya University) and Todd A. Wyatt (University of Nebraska), respectively.

### Synthesis of *N*^ε^-(M2FA)-L-lysine (M2FA-lysine) adducts

MDA was generated as previously reported^49^. M2FA-lysine was synthesized as described in Figure S1a. 4 mM of Boc-lysine, 4 mM of formaldehyde, and 8 mM of MDA were dissolved in PBS. The reaction mixture was incubated at 37 °C for 3 days. During this time, a yellowish color developed. M2FA-Boc-lysine was purified by HPLC using an Agilent 1200 HPLC system (Agilent, Santa Clara, CA) with a Poroshell 120 EC-C18 column (Agilent, 4.6 × 50 mm, 2.7 um) and isocratic elution by 0.1% formic acid and 25% acetonitrile in water at a flow rate of 1 ml/min and UV detection at 264 nm. Fractions (3 mL) were collected by an autosampler, with both the tray and fraction collector chamber maintained at 4 °C. The retention time of the fraction containing M2FA-Boc-lysine was determined, and a 2.5 to 3 min fraction of the mobile phase eluate containing M2FA-Boc-lysine was collected automatically. The collected fractions were evaporated and further incubated with 150 µL of 100% trifluoroacetic acid (TFA) overnight to remove the Boc protecting group. Following evaporation, the fractions were dissolved in HPLC-grade water for subsequent HPLC purification. M2FA-lysine was purified by the HPLC system described above using a gradient elution program as follows: eluant A, 0.1% formic acid in water (A); eluant B, acetonitrile (B) starting at 4% B and held at 4% B for 1.7 min, followed by increasing linearly to 25% B over 0.5 min and held at 25% B for an additional 2 min before re-equilibration with 4% B for 4 min. The retention time of M2FA-lysine was determined, and a 2.5 to 3.0 min fraction containing M2FA-lysine was collected automatically. The fractions were evaporated and used for LC-MS and NMR characterization, and BSA/KLH-conjugation.

### Synthesis of *N*^ε^-M2FA-6-ACA (M2FA-6ACA) adducts

M2FA-6ACA was synthesized as described for the M2FA-lysine preparation with some modifications (Fig. S2a). 4 mM of 6-ACA, 4 mM of acetaldehyde, and 8 mM of MDA were dissolved in PBS. The reaction mixture was incubated at 37 °C for 3 days. M2FA-6ACA was purified by HPLC as described above using a gradient elution program as follows: 0.1% formic acid in water (A) and acetonitrile (B) at 2% B linearly increasing to 5% B over 2 min, then increased linearly to 25% B over 0.5 min, held at 25% for an additional 3.5 min, linearly decreased to 2% B over 0.5 min and maintained at 2% B for 3.5 min. The retention time of M2FA-6ACA was determined, and a 6.0 to 6.2 min fraction of the eluate containing M2FA-6ACA was collected automatically. The fractions were evaporated and used for LC-MS, NMR characterization, and BSA/KLH-conjugation.

### Synthesis of *N*^ε^-M2AA-6-ACA (M2AA-6ACA) adducts

M2AA-6ACA was synthesized as previously described^23^. 4 mM of 6-ACA, 4 mM of acetaldehyde, and 8 mM of MDA were dissolved in PBS. The reaction mixture was incubated at 37 °C for 3 days. M2AA-6ACA was purified by HPLC as described above for M2FA-6ACA. The retention time of M2AA-6ACA was determined, and a 6.4 to 7.1 min fraction of the eluate containing M2AA-6ACA was collected automatically. The fractions were evaporated and used for LC-MS, NMR characterization, and BSA-conjugation^23^.

### LC-MS analysis

M2FA-lysine, M2AA-6ACA^23^, and M2FA-6ACA were characterized with an Agilent Technologies 1200 HPLC and 6520 Accurate Mass Q-TOF mass spectrometer (Santa Clara, CA). Products were injected on a Waters Acquity CSH Fluoro-phenyl column, 2.1 mm × 100 mm, 1.7 µm particle size. Mobile phase was delivered isocratically at 0.2 mL/min using 70% water containing 0.1% formic acid and 30% acetonitrile. Solvent flow was diverted to waste for the first 1.2 min of the analysis. Mass spectrometer parameters were set to the following values: positive ionization mode, capillary voltage of 3500 V, nebulizing gas pressure of 40 psi, drying gas temperature of 300 °C, drying gas flow of 12 l/min, and fragmentor voltage of 150 V. Scans from *m/z* 100 to *m/z* 1700 were acquired at a rate of 1 scan/s in the high-resolution, low-mass instrument state. Reference masses used for real-time mass axis adjustment were purine, *m/z* 121.050873 and HP-0921, *m/z* 922.009798.

### NMR Spectrometry

^1^H NMR spectra were recorded on a Varian INOVA 400 spectrometer at 400 MHz. Chemical shifts are reported in ppm relative to TMS.

**M2FA-lysine** (Figs 1b and 2a, and Fig. S1a and b).

C_13_H_18_N_2_O_4_:UV (ddH_2_O):  λ_max_ = 260 nm. ESI-MS:  *m*/*z* 267.1331 MH^+^.^1^H NMR (400 MHz, ACN-*d*_3_):  δ 9.26 (singlet, 2H, C*H*O), 7.32 (singlet, 2H, dihydropyridine-*H2,H6*), 3.20 (triplet, 2H, NC6*H*_2_), 2.80 (s, 2H, dihydropyridine-*H4*), 1.51-1.83 (m, 4H), 1.13-1.37 (m, 2H), (C3*H*_2_, C4*H*_2_, C5*H*_2_), ppm.

**M2FA-6ACA** (Fig. 2b; Fig. S2a and b).

C_13_H_17_NO_4_:UV (ddH_2_O):  λ_max_ = 260 nm. ESI-MS:  *m*/*z* 252.1220 MH^+^, *m/z* 250.1086 M^−^. ^1^H NMR (400 MHz, DMSO-*d*_6_):  δ 9.24 (singlet, 2H, C*H*O), 7.34 (singlet, 2H, dihydropyridine-*H2,H6*),, 3.47 (triplet, *J* = 7.2 Hz, 2H, C6*H*_2_), 2.87 (s, 2H), 2.23 (triplet, *J* = 7.2Hz, 2H, C2*H*_2_COOH),1.62 (quintet, *J* = 7.3Hz, 2H), 1.53 (quintet, *J* = 7.5Hz, 2H), 1.29 (m, 2H) ppm.

### Fluorescence measurements

Fluorescence properties of M2AA-6ACA and M2FA-6ACA were characterized using a CLARIOstar microplate reader (BMG LABTECH) equipped with a scanning mode of continuous adjustable wavelength (320-850 nm). Fluorescence measurements were also performed for M2FA-Lys/-6ACA and M2AA-Lys/-6ACA and the BSA/KLH conjugate complexes using a FLx800 microplate fluorescence reader (Bio-Tek) with excitation at 360/40, 400/10, and 485/20 nm and emission measured at 460/40, and 528/20 nm.

### Preparation of antigens

Fifty nmol of M2FA-Lys, M2FA-6ACA, or M2AA-6ACA^23^ were coupled to either 2 mg of BSA or KLH using the Imject EDC mcKLH Spin Kit, according to the manufacturer’s directions (Fig. S3). Identical amounts of purified M2FA- or M2AA-6ACA were also conjugated with magnetic beads (MB) (Dynabeads M-270 Amine). 1-Ethyl-3-(3-dimethylaminopropyl)-carbodiimide (EDC)-mediated amide formation was used for conjugation between M2AA/M2FA epitopes containing a carboxyl moiety and either BSA, KLH, or MB. The antigens conjugated with BSA/KLH were purified by extensive dialysis in PBS and sterile filtered (0.2 µm, Fisher Scientific). Pure antigens were referred to as pM2FA-Lys-BSA, pM2FA-6ACA-BSA, pM2FA-6ACA-KLH, pM2FA-6ACA-MB, pM2AA-Lys-BSA, pM2AA-6ACA-BSA, pM2AA-6ACA-KLH, and pM2AA-6ACA-MB.

### Polyclonal antibody preparation

A polyclonal antibody against pM2FA-Lys was raised in rabbits by the Pocono Rabbit Farm and Laboratory (Canadensis, PA) using pM2FA-6ACA-BSA as the antigen. The immunization was performed under an approved Institutional Animal Care and Use Committees (IACUC) permit. Briefly, rabbits were intradermally injected with ~200 µg antigen per injection with complete Freund’s adjuvant. Two, four, eight, and eleven weeks later, 100 µg antigen in incomplete Freund’s adjuvant was injected intradermally. The rabbits were terminally bled at 13 weeks from the initial immunization. After purifying the IgG fraction from serum using a Protein A column, the anti-M2FA IgG antibody was further purified by affinity purification using covalently bound M2FA-6ACA and CarboxyLink Kit. Both the polyclonal rabbit IgG antibody and the immunoaffinity-purified antibody were utilized for competitive ELISA.

### Animal treatment, blood collection, and serum preparation

All mouse experiments were performed using protocols (16-153 and 16-015) approved by the IACUC of UNC in accordance with federal guidelines. *ApoE* ^−/−^ mice on a C57BL/6 background and their wild-type controls were obtained from The Jackson Laboratory. For addressing the immunogenicity of M2FA-Lys, 6-month aged C57BL/6 male mice (3 mice per group) were used. The mice were treated intraperitoneally with BSA, or pM2FA-Lys-BSA (17 fluorescence units [measured FLx800, 360 nm excitation/460 nm emission] per 100 µg BSA in 300 µL PBS) once a week for 6 weeks without adjuvant. Seven days after the final injection, mice were euthanized by CO_2_ euthanasia. Blood was collected from the abdominal vein for serum sample collection. Plasma samples treated with EDTA were stored at −70 °C until use. To study the M2FA and M2AA antibody titers of intact female C57BL/6 mice at different ages, 2- to 19-month old C57BL/6 male mice (3 mice per group) were utilized for quantitating levels of antibody against M2FA-6AC-BSA. Blood samples were collected from the abdominal vein, followed by serum or plasma separation. Each sample was then diluted 320 fold with 1% BSA in PBS for analysis with ELISA. For studying the association between an increase in M2FA antibody titer and atherosclerosis, three-month-old C57BL/6 and *ApoE* ^−/−^ male mice (4 mice per genotype) were utilized for quantitating levels of antibodies against M2FA. Blood samples were collected from either the maxillary vein or abdominal vein, followed by serum separation.

### ELISA for determining anti-M2FA and anti-M2AA antibody

Either pM2FA-/pM2AA-6ACA-BSA/KLH (50 µL/well) was seeded on the wells in the 96-well plates (Corning Incorporated, Kennebunk, ME) at 4 °C overnight. After washing, followed by blocking with 3% BSA in PBS, 50 µL of antibody or serum/plasma samples serially diluted or diluted 1:320 ratio by 1% BSA in PBS were incubated at 4 °C overnight. After washing each well, peroxidase-labeled secondary antibodies were incubated for 1 hours. After washing the wells, the 3,3′,5,5′-Tetramethylbenzidine (TMB)/H_2_O_2_ substrate was added to all the wells and kept at room temperature for 30 min. The plates were then read with a plate reader (Vmax Kinetic Microplate Reader, Molecular Devices, Sunnyvale, CA) at a wavelength of 650 nm. Combination of the primary and secondary antibodies and serum/plasma samples used for the ELISA were as follows: 1] mouse: mouse monoclonal anti-M2AA antibody (1F83) (moMoAb-1F83)^24^, mouse serum/plasma, EnVision + Single Reagents anti-mouse (IgG)-HRP (Code K4001, Dako North America, Inc., Carpentaria, CA), and goat anti-mouse IgM antibody with HRP (Thermo Fisher Scientific); 2] rabbit: rabbit polyclonal anti-M2FA antibody (rabPoAb-M2FA), anti-M2AA antibody ([2]) (rabPoAb-M2AA), EnVision + Single Reagents anti-rabbit-HRP (Code K4001, Dako North America, Inc.) and goat anti-rabbit IgG (H + L) antibody with HRP (Thermo Fisher Scientific); 3] human: human serum/plasma, and goat anti-human IgG (H + L) antibody with HRP. ELISA for determining anti-M2FA antibody titers of serum samples obtained from mice immunized with pM2AA-lysine-BSA or BSA was performed using pM2FA-6ACA-KLH and supernatant of 5% casein in PBS instead of 3% BSA.

### Competitive ELISA

Either M2FA–6ACA or M2AA-6ACA was conjugated with Dynabeads M-270 Amine using ECD for 2 hours at room temperature followed by mashing. After blocking with 1% BSA/PBS solution, primary antibody was incubated with magnetic beads coated with M2FA or M2AA for 2 hours at room temperature. After centrifugation, the supernatant was applied non-competitive ELISA using pM2FA-6ACA-BSA coated plates.

### Histology

Mice were euthanized by CO_2_ asphyxiation. The hearts were dissected and post-fixed overnight at room temperature in zinc-based fixative containing 0.5% zinc chloride, 0.5% zinc acetate, and 0.05% calcium acetate in 0.1 M Tris base buffer. Zinc-fixed, paraffin-embedded tissues were sectioned and mounted on glass slides. The sections from the aortic valve area were stained with hematoxylin and eosin (H&E) and M2FA immunohistochemistry. For immunostaining, paraffin sections were deparaffinized, rehydrated, blocked with 1% BSA (in PBS) at room temperature for 10 minutes. The specimens were then incubated overnight at 4 °C with affinity-purified polyclonal rabbit antibodies specific for M2FA at 6.25 µg/mL in 1% BSA (in PBS) at 4 °C overnight. After washing, endogenous peroxidase activity was blocked by exposure to 3% H_2_O_2_ in deionized water for 10 min. M2FA-protein adducts were detected indirectly with a horseradish peroxidase-conjugated secondary anti-rabbit antibody (polymer reagent, DAKO) at room temperature for 1 hour. Primary and secondary antibody interactions were visualized by using 3,3′-diaminobenzidine (DAB) as the chromogen.

### Human MPO -mediated M2FA formation

We utilized the MPO-H_2_O_2_-halide system, as reported previously^21^ with some modifications. Glycine (1 mM), human MPO (50 nM), H_2_O_2_ (1 mM) were incubated in PBS for 1 hour at 37 °C followed by microcon-30 purification. The filtrate was further incubated with MDA (2 mM) and 6-ACA (1 mM) for 3 days at 37 °C. The reactant was applied to LC-MS analysis.

### Statistical analysis

The antibody values are indicated as mean ± standard deviation (SD) of the mean. The statistical differences between more than two groups were analyzed by using 1-way ANOVA followed by Tukey’s multiple comparison tests. The statistical differences between the two groups (*ApoE* ^−/−^ vs. wild-type mice) were evaluated by unpaired Student t-tests. A p-value < 0.05 was considered significant.

### Data availability

The datasets generated and/or analyzed during the current study are available from the corresponding author on reasonable request.

## Electronic supplementary material

Supplemental Results
