# Supplementary material for: Evidence that endogenous formaldehyde produces immunogenic and atherogenic adduct epitopes
Source: Sci Rep. 2017 Sep 7;7:10787. doi: 10.1038/s41598-017-11289-8 (PMC5589919; doi:10.1038/s41598-017-11289-8)
Supplement: Supplementary file 1 — Supplemental Results [file 41598_2017_11289_MOESM1_ESM.doc]

**Evidence that endogenous formaldehyde produces immunogenic and atherogenic adduct epitopes**

Jun Nakamura, Takasumi Shimomoto, Leonard B. Collins, Darcy W. Holley, Zhenfa Zhang, Jenna M. Barbee, Vyom Sharma, Xu Tian, Tomohiro Kondo, Koji Uchida, Xianwen Yi, Diana O. Perkins, Monte S. Willis, Avram Gold, & Scott J. Bultman

**Supplemental Results**

**Figure S1.**

**a.**

**
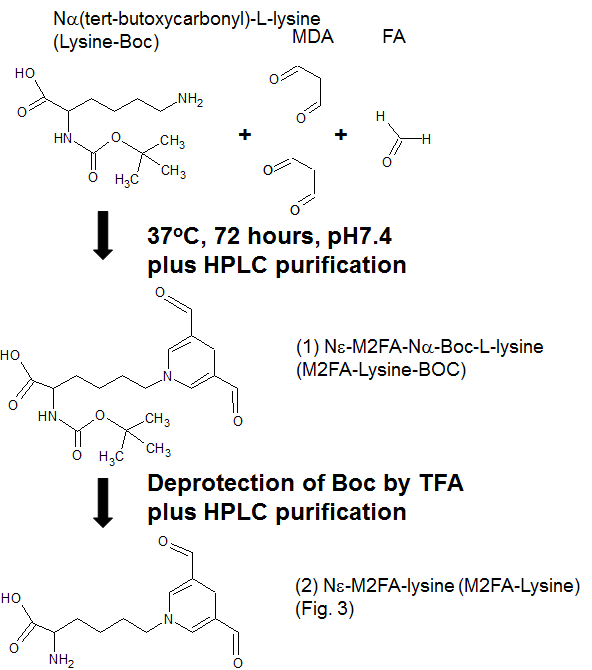
**

**b.**

**
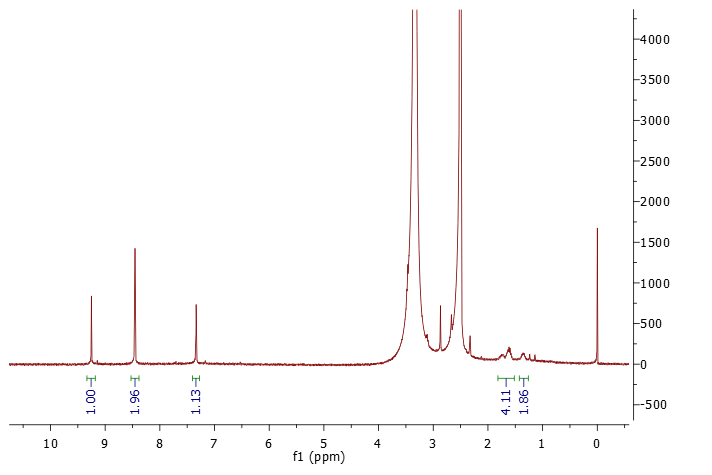
**

**M2FA-lysine preparation and NMR results.** (**a**) Boc-lysine, acetaldehyde, and MDA were incubated at 37^o^C for 3 days. M2FA-Boc-lysine was purified by HPLC system. The collected fractions were evaporated and further incubated with trifluoroacetic acid (TFA) to remove the Boc protecting group. M2FA-lysine was then purified by the HPLC system. (**b**) ^1^H NMR (DMSO-d_6_, 400 MHz): S-2-amino-6-(3,5-diformyl-pyridin-1(4H)-yl)hexanoic acid (M2FA-lysine)

**Figure S2.**

**a.**

**
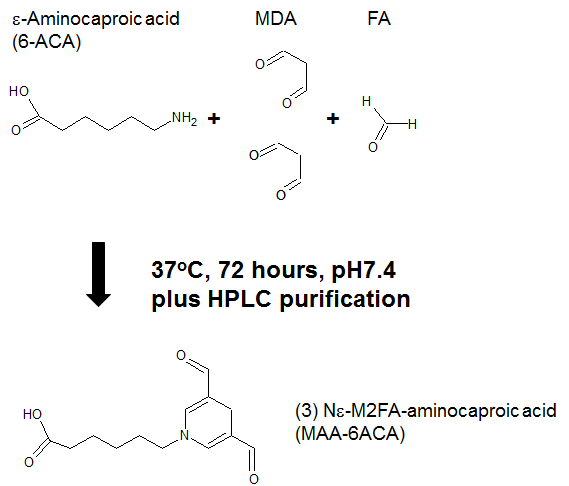
**

**b.**

**
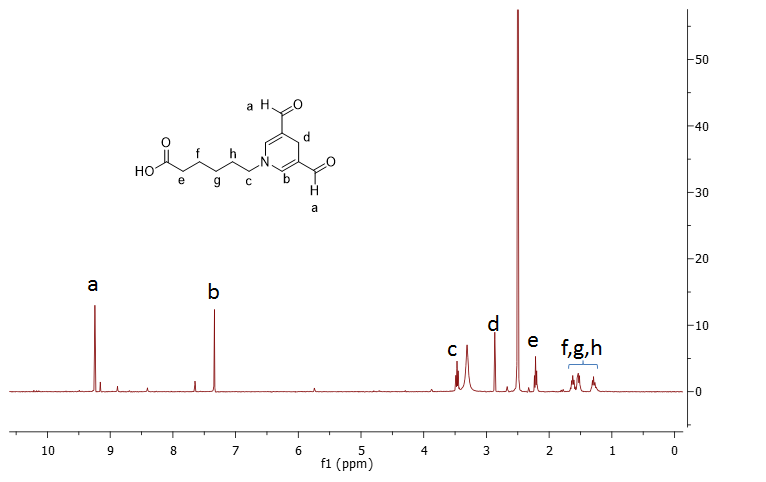
**

**MAA-6ACA preparation and NMR results**: (**a**) 6-ACA, acetaldehyde, and MDA were incubated at 37^o^C for 3 days. MAA-6ACA was purified by HPLC. **(b)** ^1^H NMR (DMSO-d_6_, 400 MHz): 6-(3,5-diformyl-pyridin- 1(4H)-yl)hexanoic acid (M2FA-6ACA).

**Figure S3.**

**
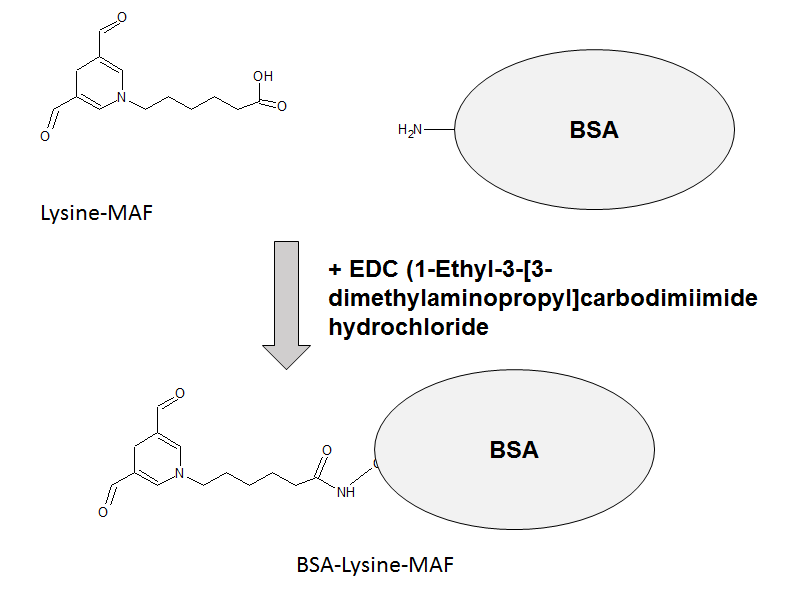
**

**Preparation of pM2FA-6ACA-BSA complex.** Pure M2FA-6ACA was coupled to BSA using the Imject EDC mcKLH Spin Kit. 1-Ethyl-3-(3-dimethylaminopropyl)-carbodiimide (EDC) -mediated amide formation was used for conjugation between pure M2FA-6ACA containing a carboxyl moiety and BSA.

**Figure S4.**

**a. b.**


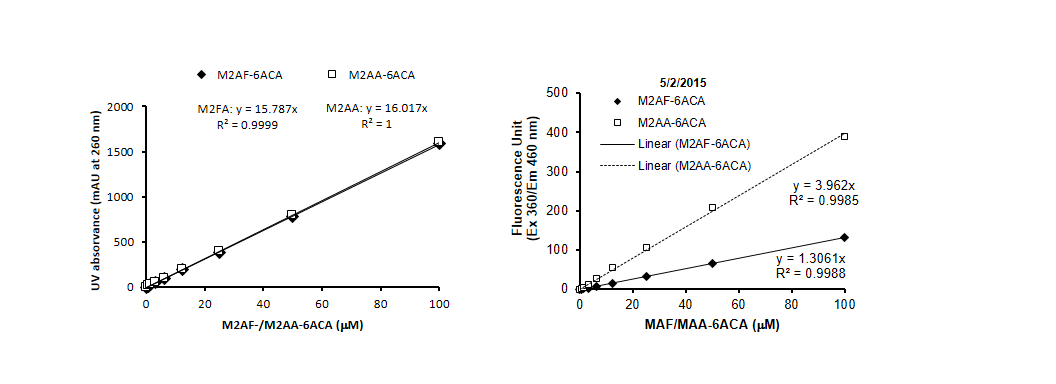


**Standard curve for M2FA-6ACA and M2AA-6ACA.** Standard curve for M2FA-6ACA and M2AA-6ACA obtained using HPLC-UV (260 nm) (**a**) and fluorescence plate reader (**b**).

**Figure S5.**


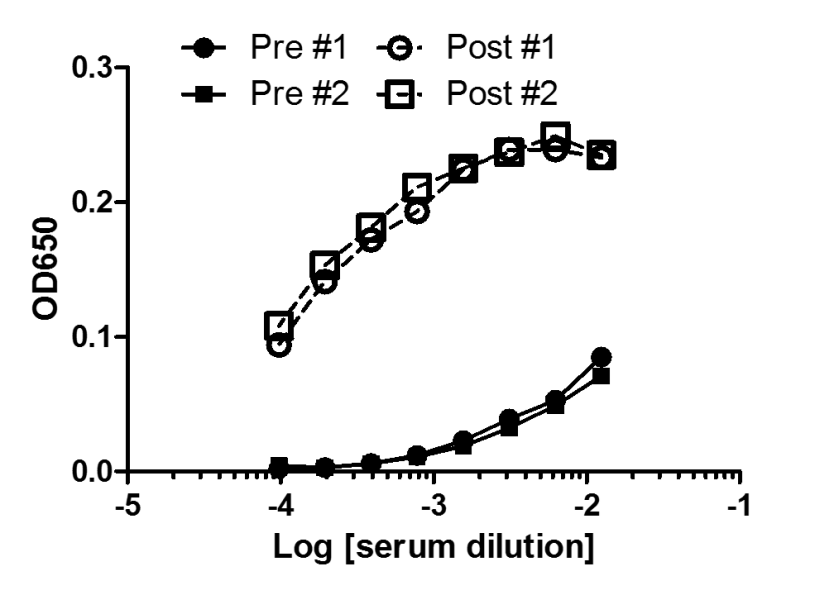


**Polyclonal antibody titer in rabbits immunized with M2FA-6ACA-BSA in the presence of adjuvant.** Two rabbits (#1 and #2) were immunized with M2FA-6ACA-BSA in the presence of adjuvant. The IgG antibody titers towards M2FA-6ACA before and after the immunization were detected using M2FA-6ACA-KLH-coated plates. The anti-M2FA antibody titers were clearly increased in M2FA-6ACA-BSA-immunized rabbits compared to those in the pre-immunized rabbits. Error bars represent SD.
